# FTLD‐TDP versus LATE‐NC: The UCSF Neurodegenerative Disease Brain Bank Experience

**DOI:** 10.1002/alz70855_106279

**Published:** 2025-12-24

**Authors:** D. Luke Fischer, Salvatore Spina, Bruce L. Miller, William W. Seeley, Lea T. Grinberg

**Affiliations:** ^1^ Memory and Aging Center, Weill Institute for Neurosciences, University of California, San Francisco, San Francisco, CA, USA; ^2^ Memory and Aging Center, UCSF Weill Institute for Neurosciences, University of California, San Francisco, San Francisco, CA, USA; ^3^ Department of Pathology, University of California, San Francisco, San Francisco, CA, USA; ^4^ Department of Neurology, Memory and Aging Center, University of California San Francisco, San Francisco, CA, USA

## Abstract

**Background:**

Similarities between frontotemporal lobar degeneration with TDP‐43 (FTLD‐TDP) and limbic‐predominant, age‐related TDP‐43 encephalopathy neuropathologic change (LATE‐NC), including some shared circuitry and similar inclusion morphologies by histology, have sparked debates about whether they represent a spectrum of the same disease. We reviewed cases from the UCSF Neurodegenerative Disease Brain Bank, which specializes in FTLD‐TDP, and contrasted them with prior reports to examine whether Type A and LATE‐NC inhabit a spectrum or are distinct diseases.

**Method:**

Patients were diagnosed with FTLD‐TDP (*N* = 148) and classified into types A‐D or deemed unclassifiable (usually due to mixed A and B features, or too sparse to classify); another 42 were classified as LATE‐NC and staged (1‐3). First, we compared the demographic, clinical, and genetic features of all FTLD‐TDP subtypes to LATE‐NC cases, including FTLD‐TDP cases with intermediate‐to‐high AD neuropathological changes (ADNC). We then focused specifically on TDP‐Type A and Type U cases, with or without coexisting AD pathology, and compared them to LATE‐NC cases due to their similar morphological inclusion pattern. Next, because LATE‐NC usually has ADNC, we contrasted TDP‐Type A or U cases with intermediate or high ADNC to LATE‐NC Stage 3. Lastly, four blinded raters assessed the middle frontal gyrus in all Type A and LATE‐NC Stage 3 cases to evaluate whether routine diagnostic examination of this region—commonly analyzed for TDP‐43 in most brain banks—can effectively differentiate Type A from LATE‐NC Stage 3.

**Result:**

FTLD‐TDP Type A has younger symptom onset, age at death, and shorter disease duration than LATE‐NC, whereas FTLD‐TDP+AD cases are in between. LATE‐NC was diagnosed only in patients with sporadic disease, whereas Type A was often genetic. FTLD‐TDP‐A was associated with FTD syndromes, whereas LATE‐NC often correlated with an AD‐associated (amnestic) syndrome. Diagnosis using only middle frontal gyrus to assess TDP‐43 proteinopathy generally was able to differentiate cases with finalized, blinded ratings and agreement analyses pending.

**Conclusion:**

FTLD‐TDP Type A and LATE‐NC share some histological features, but they appear to be distinct entities in terms of clinical, genetic, and regional disease burden. Keeping separate diagnostic terms seems appropriate.

